# Ceramic Fiber-Reinforced Polyimide Aerogel Composites with Improved Shape Stability against Shrinkage

**DOI:** 10.3390/gels10050327

**Published:** 2024-05-10

**Authors:** Wanlin Shi, Mengmeng Wan, Yating Tang, Weiwang Chen

**Affiliations:** 1 School of Mathematics and Physics, North China Electric Power University, Beijing 102206, China; 50902433@ncepu.edu.cn; 2 Key Laboratory of Civil Aviation Thermal Hazards Prevention and Emergency Response, Civil Aviation University of China, Tianjin 300300, China

**Keywords:** polyimide, aerogels, ceramic fibers, shrinkage, shape stability, thermal insulation

## Abstract

Polyimide (PI) aerogels, renowned for their nano-porous structure and exceptional performance across a spectrum of applications, often encounter significant challenges during fabrication, primarily due to severe shrinkage. In this study, we innovatively incorporated ceramic fibers of varying diameters into the PI aerogel matrix to enhance the shape stability against shrinkage. The structure of the resulting ceramic fiber-reinforced PI (CF-PI) aerogel composites as well as their performance in thermal decomposition, thermal insulation, and compression resistance were characterized. The results revealed that the CF-PI aerogel composites dried by supercritical ethanol achieved greatly reduced shrinkage as low as 5.0 vol.% and low thermal conductivity ranging from 31.2 mW·m^−1^·K^−1^ to 35.3 mW·m^−1^·K^−1^, showcasing their excellent performance in shape stability and thermal insulation. These composites also inherited the superior residue-forming ability of ceramic fibers and the robust mechanical attributes of PI, thereby exhibiting enhanced thermal stability and compression resistance. Besides, the effects of different drying conditions on the structure and properties of CF-PI aerogels were also discussed. The coupling use of supercritical ethanol drying with the addition of ceramic fibers is preferred. This preferred condition gives birth to low-shrinkage CF-PI aerogel composites, which also stand out for their integrated advantages include high thermal stability, low thermal conductivity, and high mechanical strength. These advantages attribute to CF-PI aerogel composites substantial potential for a wide range of applications, particularly as high-performance thermal insulation materials for extreme conditions.

## 1. Introduction

It is well known that aerogels are a kind of highly porous material with exceedingly low density and high specific surface area, manifesting huge potentiality in various applications [1]. Among them, polyimide (PI) aerogels are very attractive [2]. They not only inherit the excellent thermal stability and mechanical strength of PI, but also gain the advantages of an aerogel. PI aerogels can be available as blocks, fibers, films, powders, etc., and have obtained extensive attention for applications in the fields of aerospace, thermal insulation, dielectric protection, and so on [3,4,5,6,7]. 

The sol–gel method is usually used to produce a variety of nanostructured materials, as it operates under mild and low-cost conditions [8]. Based on this method, PI aerogels can be obtained by in situ polymerization of precursor polyamic acid (PAA) solutions [9]. This involves the nucleophilic substitution of dianhydrides and diamines, yielding PAA that must be further dehydrated to convert into PI. The process of dehydration can be executed via thermal imidization at high temperatures or chemical imidization with the help of a catalyst and dehydrating agent. In addition, a series of subsequent steps, such as aging, solvent exchange, and drying, are also required. The drying can be supercritical drying, freeze drying, atmospheric drying, etc. [10]. However, the preparation of PI aerogels is often troubled by severe shrinkage [11,12]. Many studies, therefore, have made efforts to reduce shrinkage, which primarily fall into two categories: chemical modifications and physical support [13]. In terms of chemical modifications, cross-linkers such as poly(maleic anhydride), dopamine-functionalized carbon nanotubes (CNT-NH_2_), aminated SiO_2_ nanoparticles (NH_2_-SiO_2_), etc., have been employed [14,15,16,17,18,19]. For example, Lin et al. used an excess amount of 1,3,5-benzenetricarbonyl trichloride (BTC) to prepare cross-linked PI aerogels, which effectively reduced the shrinkage of PI aerogels in the preparation process from above 90% to about 40% [15]. Wang et al. used CNT-NH_2_ as the crosslinking agent to prepare PI aerogels, which reduced the shrinkage from 48.4% to 17.9–19.0% [18]. Liang et al. used another crosslinker, 4,4-oxydianiline (ODA)-modified graphene oxides (m-GO), and obtained PI/m-GO aerogels with a shrinkage of about 26%, less than the shrinkage of 34% in the PI aerogel cross-linked with 1,3,5-triaminophenoxybenzene (TAB) [19]. These studies did lead to a significant reduction in the shrinkage of PI aerogels. Nevertheless, the reduced shrinkage is still not satisfactory, and the process itself is quite challenging as chemical modification always requires many additional operations to control the reaction. In contrast, physical support offers a more cost-effective, eco-friendly, and straightforward approach [20,21,22,23]. For instance, Zuo et al. effectively reduced the shrinkage from 27.8 vol.% to 21.1 vol.% by incorporating graphene and montmorillonite into PI aerogels [22]. Kantor et al. also observed a significant reduction in shrinkage upon the addition of silica aerogel powder into the PI aerogel matrix [24]. The volume shrinkage of the aerogel-in-aerogel composite with 28 wt.% silica aerogel powder added was reduced by 40% due to the high shape preservation capability of silica aerogel. Likewise, Zhu et al. incorporated a glass fiber mat for PI aerogels during preparation and reduced the shrinkage from 57.9 vol.% to 16.5 vol.% [23]. These results revealed that physical support can effectively reduce shrinkage to a certain extent. However, the remaining shrinkage is still quite large and may severely change the size and shape of PI aerogels, especially during the process of preparation.

To solve the in-preparation shrinkage issue of PI aerogels, in this study, we utilized ceramic fibers of varying diameters for better support and obtained a series of ceramic fiber-reinforced polyimide (CF-PI) aerogel composites. The structure and properties of the resulting CF-PI aerogel composites were characterized. It was confirmed that the incorporation of ceramic fibers significantly enhances the shape stability of PI aerogels to resistant shrinkage and deformation during preparation. With the aid of supercritical ethanol drying, the prepared CF-PI aerogel composites also excelled in thermal insulation, mechanical strength, and thermal endurance, presenting great potential for diverse applications.

## 2. Results and Discussion

### 2.1. Morphology and Structure

**Figure 1:**
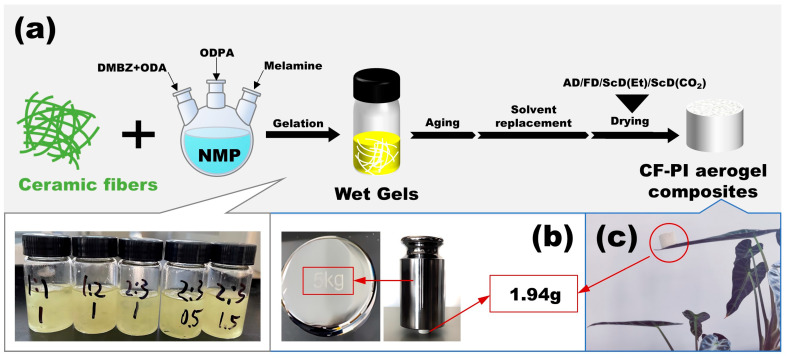
Schematic illustration of the preparation of CF-PI aerogel composites (**a**). The muscular and ultralight CF_1_-PI_1-1_-ScD (Et) can easily support a counterpoise of 5 kg (**b**), and can be effortlessly lifted by a single leaf without causing it to bend (**c**).

**Figure 2:**
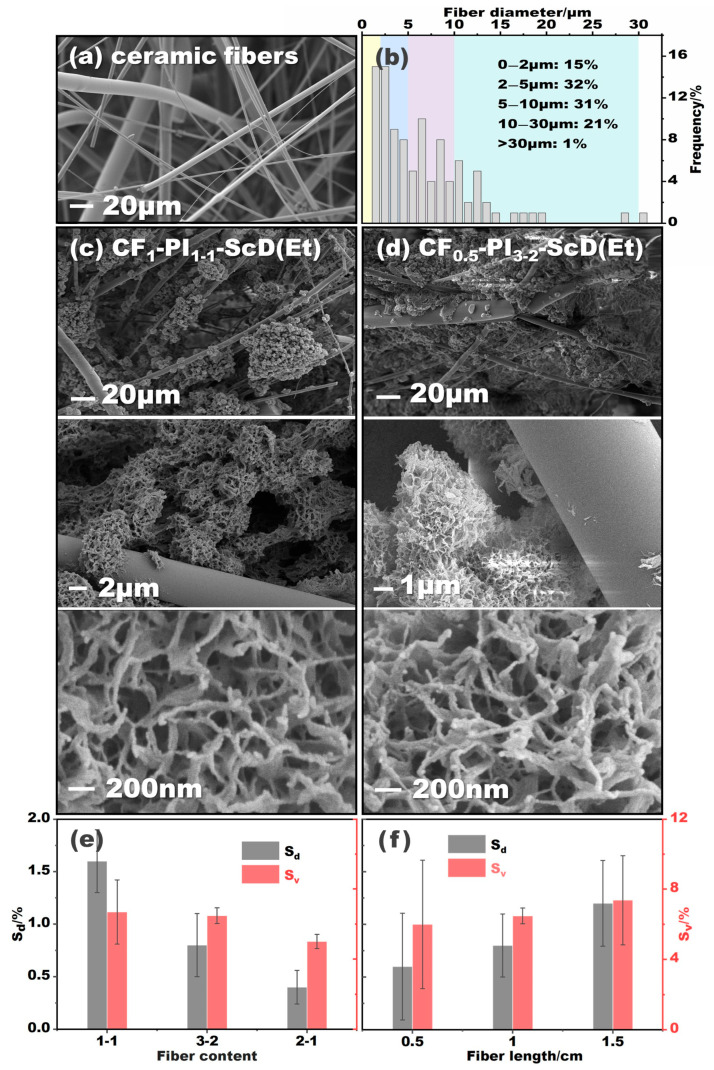
SEM images (**a**) and diameter distribution (**b**) of the ceramic fibers of varying diameters. The microstructure of representative CF-PI aerogel composites dried by supercritical ethanol (**c**,**d**), and the variations in shrinkage of the CF-PI-ScD (Et) aerogel composites with fiber content (**e**) and fiber length (**f**).

The CF-PI-ScD (Et) aerogel composites exhibit a uniform cylindrical shape, as depicted in Figure 1c, similar to our previously reported neat PI aerogels. Figure 2a illustrates the ceramic fibers used for support, which display a range of diameters. Above 50% of the ceramic fibers have diameters exceeding 5 μm, while about 15% are finer than 2 μm. This variation in diameter is strategic: the fibers of larger diameters act as structural frameworks, similar to tree trunks, while the finer and more flexible fibers serve as branches, enhancing the integration with the aerogel matrix. The SEM images in Figure 2c,d reveal a network of ceramic fibers, which are randomly distributed to provide structural support within the nano-porous aerogel. In the CF-PI-ScD (Et) aerogel composites, these fibers form a supportive framework, filled with PI aerogel as the functional component. This interwoven structure, featuring ceramic fibers of varying sizes, is expected to significantly reduce the shrinkage. This is indeed the case. The results in Figure 2e,f and Table 1 show that the S_d_ and S_v_ of CF-PI-ScD (Et) aerogel composites during preparation are only 0.4–1.6% and 5.0–7.4%, respectively, with shrinkage diminishing as fiber content increases or fiber length decreases. For CF-PI-ScD (Et) aerogel composites with a higher fiber content, shrinkage is reduced as the denser supporting framework enhances the stability of the composites. The data in Table 1 show that increasing the fiber content from a 1:1 to a 2:1 ratio leads to a steady decrease in S_v_ from 6.7% to 5.0%. The reason why shorter fibers lead to less shrinkage is simple: their bending resistance increases with reduced length, providing superior anti-shrinkage support [23]. Additionally, shorter fibers disperse more uniformly in precursor solution, leading to a more homogeneous structure and avoiding local vacancy or fiber aggregation. As a result, the CF-PI-ScD (Et) aerogel composites with shorter fibers incorporated show better performance against shrinkage. An increase in fiber length from 0.5 cm to 1.5 cm therefore results in a gradual increase in S_v_ from 6.0% to 7.4%. Taken together, however, the shrinkage of these complexes is not very different from each other. The porous structure of the PI aerogel matrix in different composites is therefore similar to each other, as shown in Figure 2c,d. Notably, the shrinkage observed here is significantly less than that reported in previous studies [25,26]. It should be noted that, due to the harsher condition of supercritical ethanol drying, most of the PI aerogels prepared by this method tend to suffer a more severe shrinkage than those dried by supercritical CO_2_. But, this does not seem to be true for the CF-PI-ScD (Et) aerogel composites prepared in this study. They exhibit much lower shrinkage than that of the PI aerogels prepared by supercritical CO_2_ in many other studies. This is mainly due to the introduction of ceramic fibers. This indicates the feasibility and effectiveness of the current strategy, and it suggests that incorporating various-sized ceramic fibers can substantially reduce the shrinkage of PI aerogels.

### 2.2. Thermal Properties

**Figure 3:**
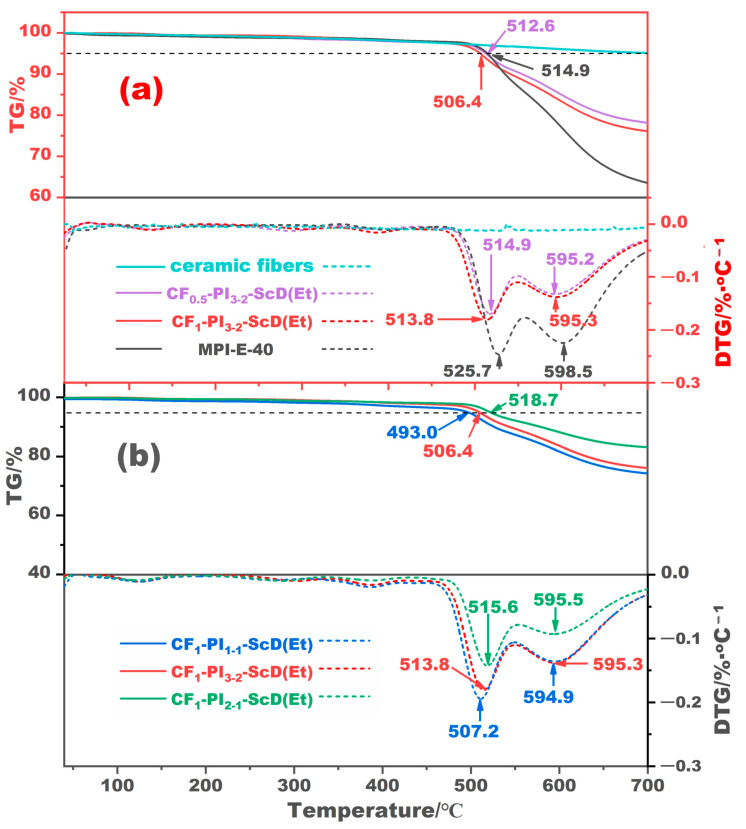
TG and DTG curves of the CF-PI-ScD (Et) aerogel composites with different fiber lengths (**a**) and fiber contents (**b**).

Figure 3a presents the TG and DTG curves of ceramic fibers, and the CF_0.5_-PI_3-2_-ScD (Et) and CF_1_-PI_3-2_-ScD (Et) aerogel composites, with the MPI-E-40 from our previous study as a reference [26]. As expected, the ceramic fibers undergo a slight weight loss over the entire temperature range. For both CF-PI-ScD (Et) aerogel composites and the MPI-E-40, they almost do not decompose before 500 °C due to the pre-baptism of supercritical ethanol drying. The difference between CF-PI-ScD (Et) and MPI-E-40 mainly lies in the post-pyrolysis residue, where the CF-PI-ScD (Et) composites perform much better in residue-forming. This enhancement is attributed to the superior thermal stability of ceramic fibers compared to PI, indicating that the incorporation of ceramic fibers not only preserves but also augments the thermal stability of the composites to some extent. Taking into account that the residue of ceramic fibers and MPI-E-40 at 700 °C is 95.1% and 63.5%, respectively, the residue of CF_0.5_-PI_3-2_-ScD (Et) and CF_1_-PI_3-2_-ScD (Et) aerogel composites at the same temperature should be 82.4%. The differences between the theoretical prediction and the experimental results are less than 6.5%. This is easy to understand because the distribution of ceramic fibers cannot be uniform everywhere.

Figure 3b illustrates the TG and DTG curves of CF-PI-ScD (Et) aerogel composites with varying fiber contents. The onset temperature of decomposition, denoted as *T*_-5wt%_, i.e., the temperature at which the composite loses 5% of its mass, is observed to rise incrementally with an increase in fiber content. Specifically, the *T*_-5wt%_ for the CF_1_-PI_2-1_-ScD (Et) aerogel composite is 518.7 °C, 12.3 °C, and 25.7 °C higher than those for CF_1_-PI_3-2_-ScD (Et) and CF_1_-PI_1-1_-ScD (Et), respectively. This trend can be predictable, as a higher fiber content correlates with a reduced PI content, requiring a higher temperature to achieve the same weight loss. Furthermore, the residue of the CF-PI-ScD (Et) aerogel composites at 700 °C also remains impressively high, ranging from 74.2% to 83.1% of the initial mass, with higher fiber content correlating with greater residue. The differences between the experimental residue and the corresponding predicted ones were also within 6.5%. These findings suggest that CF-PI-ScD (Et) aerogel composites, with their significantly reduced shrinkage, enhanced residue-forming capability, and inherent outstanding thermal stability, hold considerable promise for high-temperature applications.

**Figure 4:**
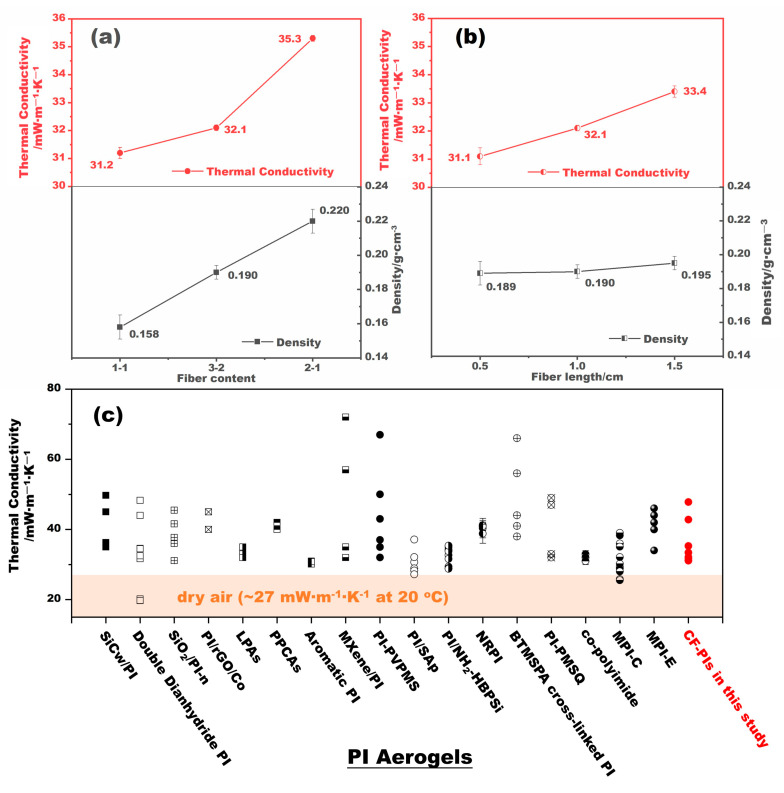
The variations in the thermal conductivity and the density of CF-PI-ScD (Et) aerogel composites with fiber content (**a**) and fiber length (**b**). The comparison of the thermal conductivity of CF-PI-ScD (Et) aerogel composites in this study with some other PI aerogels (**c**), including SiCw/PI aerogel composites [21], double dianhydride backbone PI aerogels [29], SiO_2_/PI-n aerogels [30], PI/rGO/Co aerogels [31], linear polyimide aerogels (LPAs) [32], PI-PVPMS composite aerogels (PPCAs) [32,33], aromatic PI aerogels [34], MXene/PI aerogels [35], PI/SAp aerogels [36], PI/NH_2_-HBPSi aerogels [37], nanofiber-reinforced polyimide (NRPI) aerogels [18], BTMSPA cross-linked PI aerogels [38], PI-PMSQ aerogels [39], co-polyimide aerogels [9], melamine-crosslinked polyimide aerogels dried by supercritical CO_2_ (MPI-C) [25], and ethanol (MPI-E) [26]. The SiCw, rGO, PVPMS, MXene, SAp, NH_2_-HBPSi, BTMSPA, and PMSQ given here refer to SiC whiskers, reduced graphene oxide, polyvinylpolymethylsiloxane, 2D transition metal carbides and nitrides, silica aerogel powder, amine-functionalized hyperbranched polysiloxane, bis(trimethoxysilylpropyl) amine, and polymethylsilsesquioxane, respectively.

As shown in Figure 4a, the bulk densities of the CF-PI-ScD (Et) aerogel composites increase from 0.158 g·cm^−3^ to 0.220 g·cm^−3^ as fiber content increases from 1:1 to 2:1, despite a concurrent reduction in shrinkage. The increase in density implies an increase in solid conduction channels or wider pathways per unit volume, indicating that the introduction of ceramic fibers bolsters solid-phase thermal conduction to some extent. Accordingly, when the fiber content advances from 1:1 to 2:1, the thermal conductivity of the CF-PI-ScD (Et) aerogel composites experiences a marginal yet progressive rise, from 31.2 mW·m^−1^·K^−1^ to 35.3 mW·m^−1^·K^−1^. This trend is consistent with that reported by Fei et al. [27]. If the mass ratio of ceramic fibers to the PI polymer holds constant while the fiber length changes, as Figure 4b shows, the density of the CF-PI-ScD (Et) aerogel composites remains relatively stable at approximately 0.190 g·cm^−3^. This aligns with the observations of Jaxel et al., who reported that fiber length exerts a negligible influence on the density of silica aerogel composites reinforced with cellulose fibers [28]. The thermal conductivity of CF-PI-ScD (Et) aerogel composites, however, shows a slight gradual increase from 31.1 mW·m^−1^·K^−1^ to 33.4 mW·m^−1^·K^−1^ as the fiber length increases from 0.5 cm to 1.5 cm. This is likely due to the fact that, after using short ceramic fibers, the fast channels for heat transfer along these fibers are shortened, leading to an increase in contact thermal resistance. The results suggest that, for applications requiring superior thermal insulation, the use of short fibers as supports for PI aerogels is more attractive than the use of long fibers. 

Figure 4c shows the comparison of CF-PI-ScD (Et) aerogel composites in this study with air and some other PI aerogels in terms of thermal insulation. It is apparent that the thermal conductivity of CF-PI-ScD (Et) aerogel composites, ranging from 31.1 mW·m^−1^·K^−1^ to 35.3 mW·m^−1^·K^−1^, is not much higher than that of dry air (~27 mW·m^−1^·K^−1^ at 20 °C) and is comparable to the that of the reported PI aerogels. This makes CF-PI-ScD (Et) aerogel composites quite attractive, highlighting their great potential as superior barriers for thermal insulation.

### 2.3. Mechanical Strength

**Figure 5:**
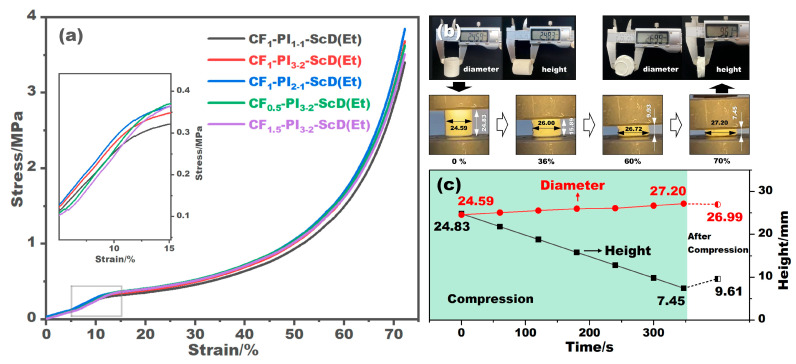
Compressive stress–strain curves of the CF-PI-ScD (Et) aerogel composites with different fiber contents and fiber lengths (**a**). Shape and dimensions of the CF_1_-PI_1-1_-ScD (Et) aerogel composite before, during, and after compression (**b**), together with the variations in its height and diameter with compression (**c**).

A sensor with a maximum capacity of 1000 N was utilized to assess the compressive behavior of CF-PI-ScD (Et) aerogel composites, with the resulting stress–strain curves depicted in Figure 5a. From these curves, the CF-PI-ScD (Et) aerogel composites show elastic deformation between 0–12%, characterized by a near-linear relationship between stress and strain. Subsequently, within the 12–50% strain range, the porous structure of the composites begins to collapse, leading to irreversible plastic deformation and a consequent plateau in the stress–strain curve, where stress increases at a slower rate. Beyond a 50% strain, the composites enter a densification phase. Figure 5b,c shows the deformation of the CF_1_-PI_1-1_-ScD (Et) aerogel composite during compression, together with the variations of its height and diameter with compression. It is obvious that the height of the aerogel composite decreases gradually during compression, resulting in a gradual but slight increase in diameter. This is mainly due to the high porosity of the PI aerogel, and because of this, CF_1_-PI_1-1_-ScD (Et) tends to destroy aerogel pores and expel the air in the pores during compression to make the aerogel composite dense without significantly expanding its diameter. Once the compressive stress is removed, the sample further shows a slight height recovery, increasing from 7.45 mm to 9.61 mm, and experiences a slight reduction in diameter, from 27.20 mm to 26.99 mm.

For the CF-PI-ScD (Et) aerogel composites with varying fiber contents, it is observed that the compressive strength increased with the increase in fiber content. As listed in Table 1, the Young’s modulus of these composites rises from 2.46 MPa to 2.77 MPa as the fiber content increases from 1:1 to 2:1. This makes sense, because the more ceramic fibers are added, the more fibers will be radially aligned to resist external forces, as these fibers are randomly distributed. Conversely, for composites with different fiber lengths, the Young’s modulus exhibits a decline with an increase in fiber length. This inverse relationship aligns with the principle that fibers of the same type but shorter lengths offer enhanced resistance to bending. This is why the Young’s modulus of CF_1.5_-PI_3-2_-ScD (Et) stands at 2.60 MPa, which is 0.03 MPa and 0.10 MPa lower than those of CF_1_-PI_3-2_-ScD (Et) and CF_0.5_-PI_3-2_-ScD (Et), respectively. These findings suggest that the incorporation of more ceramic fibers with shorter lengths can somewhat bolster the mechanical strength of composite aerogels, but the bolstering effect is limited.

### 2.4. The Effect of Drying Conditions

**Figure 6:**
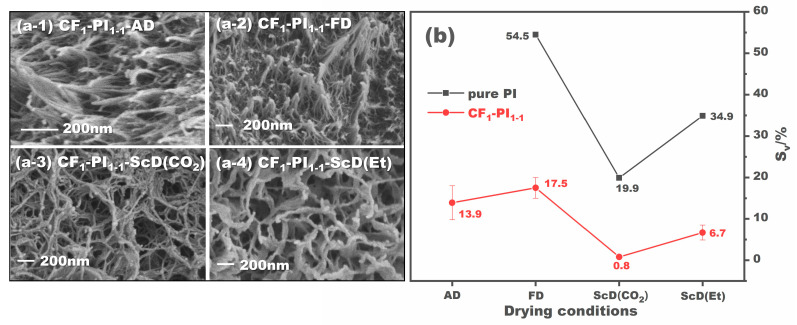
SEM images of the CF_1_-PI_1-1_ aerogel composites prepared under different drying conditions (**a**), and the volume shrinkage of these CF_1_-PI_1-1_ aerogel composites compared to pure PI aerogels (**b**).

The drying condition has a significant impact on the shrinkage of aerogels, which in turn affects their properties. In this study, we also explored the effects of different drying conditions on the structure and properties of CF-PI aerogel composites, including atmospheric drying, freeze drying, and supercritical drying with CO_2_ and ethanol. The morphology of the CF-PI aerogel composites obtained under the given drying conditions is shown in Figure 6a. It is apparent that the CF-PI aerogel composites obtained from atmospheric drying and freeze drying, i.e., the CF_1_-PI_1-1_-AD in Figure 6(a-1) and the CF_1_-PI_1-1_-FD in Figure 6(a-2), have relatively denser inner structures. This is because both atmospheric drying and freeze drying can bring PI aerogels serious shrinkage, which, despite the support of ceramic fibers, leads to a destruction and compaction of the inner porous structure. The volume shrinkage of the CF_1_-PI_1-1_-AD and CF_1_-PI_1-1_-FD aerogel composites is calculated to be as high as 13.9% and 17.5%, respectively, as shown in Figure 6b. In contrast, the porous structure of PI aerogels can be well preserved by supercritical drying. This is because supercritical drying enables the transformation of the drying medium into a supercritical state with zero surface tension, thereby preventing the collapse of the aerogel structure. Figure 6(a-3,a-4) shows the microscopic morphology of CF_1_-PI_1-1_-ScD (CO_2_) and CF_1_-PI_1-1_-ScD (Et), respectively. The CF_1_-PI_1-1_-ScD (CO_2_) appears as “stacked weeds”, while the CF-PI aerogel composite obtained from supercritical ethanol drying presents to be “clumped shrubs” with severe fiber conglutination. This should be due to the harsher condition of supercritical ethanol drying (276 °C and 8 MPa), while supercritical CO_2_ drying for preparing CF-PI-ScD (Et) aerogel composites is operated under much milder conditions of 45 °C and 7 MPa. As a result, CF-PI-ScD (Et) aerogel composites shrink more than CF-PI-ScD (CO_2_) aerogel composites. The volume shrinkage of CF_1_-PI_1-1_-ScD (Et) is calculated to be 6.7%, much higher than the 0.8% of CF_1_-PI_1-1_-ScD (CO_2_). Obviously, these two shrinkage values are much lower than those caused by atmospheric drying and freeze drying. Compared with pure PI aerogels without fiber reinforcement, the shrinkage of CF-PI aerogel composites is greatly reduced regardless of the drying condition used. For the CF-PI-ScD (Et), which we have consistently favored, its shrinkage is only one-fifth of the shrinkage of pure PI. This indicates that the addition of ceramic fibers of varying diameters can indeed significantly enhance PI aerogels’ shape stability against shrinkage.

**Figure 7:**
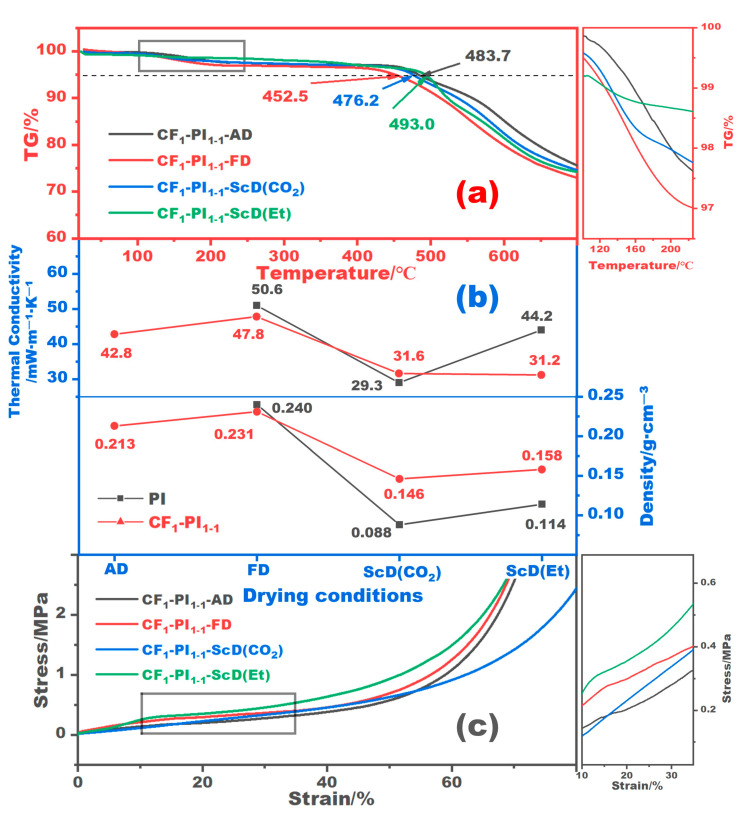
TG curves of the CF_1_-PI_1-1_ aerogel composites prepared under different drying conditions (**a**), and the variations in density and thermal conductivity of pure PI aerogels and these composites with the drying condition (**b**), as well as the compressive stress–strain curves of the CF_1_-PI_1-1_ aerogel composites (**c**).

Figure 7a illustrates the TG curves of CF_1_-PI_1-1_ aerogel composites obtained from the four drying conditions. It can be observed that the aerogel composites obtained from atmospheric drying, freeze drying, and supercritical CO_2_ drying suffer more significant weight loss around 200 °C. This is detrimental to shape stability, as the weight loss here always corresponds to the removal of possible residual solvent, moisture, etc., and because of this, the aerogel composite may shrink further. Figure 7b plots the densities and thermal conductivity of the resulting CF_1_-PI_1-1_ aerogel composites prepared under different drying conditions, as well as those of the corresponding pure PI aerogels. Obviously, the changes of these CF_1_-PI_1-1_ aerogel composites in thermal conductivity align with the changes in density. Given that their densities are inversely proportional to their shrinkage, it can be concluded that a decrease in shrinkage is beneficial to preserve the low thermal conductivity of PI aerogels. For the CF_1_-PI_1-1_-ScD (Et) aerogel composite with a volume shrinkage of 6.7%, its thermal conductivity is tested to be 31.2 mW·m^−1^·K^−1^, 16.6 mW·m^−1^·K^−1^ lower than that of the CF_1_-PI_1-1_-FD aerogel composite with a volume shrinkage of 17.5%. Note that, compared with pure PI aerogels, most of the CF-PI aerogel composites show larger densities due to the addition of ceramic fibers. But, this does not always mean a greater thermal conductivity as the thermal conductivity of an aerogel is the joint result of solid-phase thermal conduction, gas-phase thermal conduction, etc. Figure 7c shows the compressive stress–strain curves of the CF_1_-PI_1-1_ aerogel composites. It is obvious that the atmospheric-dried and freeze-dried ones, despite their relatively denser structures, do not show a significant improvement in mechanical properties compared to those prepared by supercritical drying. This is unacceptable because the shrinkage after adding the ceramic fibers is still quite large. For CF-PI aerogel composites obtained by supercritical drying with low shrinkage, those prepared by supercritical drying with ethanol show better performance in mechanical strength. This can be understood from two aspects: the first one is that the density of the CF_1_-PI_1-1_-ScD (Et) aerogel composite with a higher shrinkage of 6.7% is 0.158 g·cm^−3^, higher than the 0.146 g·cm^−3^ of the CF_1_-PI_1-1_-ScD (CO_2_) aerogel composite. That is, there are more polymer branches per unit cross-section against external forces for CF_1_-PI_1-1_-ScD (Et), shown as higher compressive strength. The second reason is that the fibrous skeletons of CF_1_-PI_1-1_-ScD (Et) are much stronger than those of CF_1_-PI_1-1_-ScD (CO_2_), as the SEM images in Figure 6a show. 

In general, even with the addition of ceramic fibers, atmospheric drying and freeze drying are still not conducive to obtaining PI aerogels with low shrinkage. As the aerogels shrink, their shape, pore structure, and properties will change accordingly, most of which are undesirable. However, if supercritical drying is selected, the volume shrinkage of CF-PI aerogel composites will be small enough to be acceptable. This holds especially in the case of composites obtained through supercritical ethanol drying, as they not only possess good shape stability against shrinkage but also stand out for their integrated advantages, including high thermal stability, low thermal conductivity, and considerable mechanical strength. These advantages make PI aerogel composites highly attractive, with broad application prospects in the field of high-temperature insulation.

### 2.5. Limitations and Future Work

This study has some limitations. For example, the diameter distribution of the ceramic fibers used for support in this study was not adjusted. That is, the diameter distribution of these fibers was as it was upon their reception. The contribution and effect of ceramic fiber diameter on reducing shrinkage in PI aerogels therefore were not considered. Besides, the CF-PI aerogel composites prepared in this study are all small-sized cylinders. For PI aerogels of larger sizes or other shapes, whether the addition of ceramic fibers would yield the same benefits against shrinkage requires further verification. In addition, the shape stability against shrinkage of CF-PI aerogel composites derived from other dianhydrides and diamines also remains unclear. In future studies, further discussions are needed to overcome these limitations. Furthermore, the role of three-dimensional tree-like fiber clusters, with thick fibers as the trunk and thin fibers as the branches, deserves special attention in reducing the shrinkage of PI aerogels.

## 3. Conclusions

In this study, PI aerogels were reinforced with ceramic fibers to enhance their shape stability against shrinkage. The findings indicate that the properties of the resulting CF-PI aerogel composites are influenced by fiber content, fiber length, and drying condition. The combination of supercritical drying with the addition of ceramic fibers is preferred to prepare low-shrinkage PI aerogel composites. By coupling the use of supercritical ethanol drying with the addition of ceramic fibers of varying diameters, a series of CF-PI aerogel composites are developed. From a general view, these composites are born as regular cylinders and are still quite light, with densities between 0.158 g·cm^−3^ to 0.220 g·cm^−3^. The diameter and volume shrinkage of CF-PI aerogel composites can be as low as 0.4% and 5.0%, respectively. It can be given that the severe shrinkage of PI aerogels during preparation is well solved, also known as greatly improved shape stability. Besides, the composites exhibit commendable thermal insulation properties with a thermal conductivity of no more than 35.3 mW·m^−1^·K^−1^, and they have improved mechanical strength, as evidenced by a Young’s modulus ≥2.46 MPa. Given these characteristics, together with their enhanced residue-forming ability and intrinsic outstanding thermal stability, CF-PI aerogel composites with low shrinkage, low densities, and low thermal conductivity hold substantial potential for high-temperature applications.

## 4. Materials and Methods

### 4.1. Materials

Dimethylbenzidine (DMBZ), 4,4′-oxydianiline (ODA), 4,4′-oxydiphthalic anhydride (ODPA), N-methylpyrrolidinone (NMP) pyridine, propionic anhydride, and melamine were purchased from Shanghai Aladdin Biochemical Technology Co., Ltd. (Shanghai, China). Ethanol was provided by Tianjin Kemiou Chemical Reagent Co., Ltd. (Tianjin, China). The reagents were used as received without further purification. The ceramic fibers of varying diameters used for physical support were aluminosilicate fibers, and they were supplied by Langfang Renheng Thermal Insulation Material Co., Ltd. (Langfang, China). The length of the ceramic fibers was controlled by cutting.

### 4.2. Preparation of CF-PI Aerogel Composites

In this study, CF-PI aerogel composites were obtained by directly adding ceramic fibers of varying diameters in PI aerogels, which were derived from ODPA and hybrid diamines of DMBZ and ODA with melamine as an economical yet effective crosslinker. Each solution for gelation was standardized at a 10 wt.% concentration. For simplicity, the resulting aerogel composites were denoted as CF*_x_*-PI*_y-z_*-N, with *x* indicating the length of the ceramic fibers in cm, *y-z* representing the mass ratio of the ceramic fibers to the PI polymer, and N standing for the drying condition used. Here, the drying conditions AD and FD refer to atmospheric drying and freeze drying, respectively, while the ScD (Et) and ScD (CO_2_) terms are short for supercritical ethanol drying and supercritical CO_2_ drying, respectively. 

The procedure for preparing CF-PI aerogel composites is depicted in Figure 1a. Taking the preparation of CF_1_-PI_1-1_-ScD (Et) as an example, initially, 0.64 g (3.20 mmol) of ODA and 0.68 g (3.20 mmol) of DMBZ were added to 20.0 mL of NMP, followed by the addition of 2.08 g (6.72 mmol) of ODPA. The mixture was stirred for a while to dissolve all the monomers to further form anhydride-terminated oligomers. Then, 3.0 mL of NMP containing 0.02 g (0.16 mmol) of melamine was added to the mixture, and the stirring was continued until the melamine was completely dissolved. After that, for chemical imidization, 6.9 mL (53.76 mmol) of propionic anhydride and 4.2 mL (53.76 mmol) of pyridine were added to the mixture. Following this, 3.40 g of pre-treated ceramic fibers of varying diameters, which had been washed, cleaned, and cut to 1 cm lengths, were added into a cylindrical glass vessel with a diameter of 25 mm. The mixture was then poured into the vessel as well, ensuring a 1:1 mass ratio of ceramic fibers to PI. To achieve a uniform dispersion of the fibers, the mixture was then shaken and subjected to ultrasonic dispersion. This resulted in a well-dispersed precursor mixture with ceramic fibers evenly distributed inside. The precursor mixture was then allowed to gel and age at room temperature for several days, yielding a wet gel. This wet gel subsequently underwent several cycles of dialysis using a stepwise decrease in the volume fraction of NMP in NMP/ethanol mixtures, set at 75%, 25%, and finally 0%. This was to remove pyridine and any residual impurities from the wet gel, preparing it for the subsequent drying process. For the final step in the preparation of CF_1_-PI_1-1_-ScD (Et), supercritical ethanol drying (276 °C and 8 MPa) was employed to transform the wet gel into an aerogel.

### 4.3. Characterization

The diameter distribution of ceramic fibers was determined by statistical analysis of over 100 randomly selected fibers. The bulk density of each CF-PI aerogel composite was determined using mass-to-volume ratios, with the average value calculated from five measurements. Both the diameter shrinkage (S_d_) and volume shrinkage (S_v_) of such aerogel composites were quantified by comparing pre- and post-drying dimensions. The morphology of the ceramic fibers, together with that of the CF-PI aerogel composites reinforced by these ceramic fibers, was observed by field emission scanning electron microscopy (FE-SEM, LEO-1530VP, ZEISS, Oberkochen, Germany). Before SEM observations, the samples were gold-sputtered for better conductivity. Pyrolysis performance was analyzed by thermogravimetric analysis (TGA, SDT650, TA, New Castle, De, USA) in N_2_ atmosphere from room temperature to 700 °C at a 10 °C·min^−1^ heating rate. Thermal conductivity was measured at room temperature with a TPS 2500S (Hot disk, Göteborg, Sweden), employing the transient plane source (TPS) method per ISO 22007-2 [40]. In the process of the thermal conductivity test, two points need to be ensured: (1) the surface of the samples should be smooth, with the round sensor probe clamped in the middle; (2) the contact area of the samples should be larger than that of the sensor probe to fully cover the sensor probe. Each sample was tested three times to obtain an average value of its thermal conductivity. Compressive performance was assessed using a WDW-1M (Zhongluchang, Jinan, China) electronic universal testing machine, with a descending speed of 3 mm·min^−1^ and a pretension load of 0.01 N. The test stopped when each sample was compressed to 70% of its initial height.

## Figures and Tables

**Table 1:** Summary of the parameters and properties of the prepared CF-PI aerogel composites.

CF-PI Aerogel Composites	Density/g·cm^−3^	Shrinkage/%		Thermal Conductivity/mW·m^−1^·k^−1^	Young’s Modulus/MPa	T_-5wt%_/°C
		S_d_	S_v_			
CF_1_-PI_1-1_-ScD (Et)	0.158 ± 0.007	1.6 ± 0.3	6.7 ± 1.8	31.2 ± 0.2	2.46	493.0
CF_1_-PI_3-2_-ScD (Et)	0.190 ± 0.004	0.8 ± 0.3	6.5 ± 0.4	32.1 ± 0.1	2.63	506.4
CF_1_-PI_2-1_-ScD (Et)	0.220 ± 0.007	0.4 ± 0.2	5.0 ± 0.4	35.3 ± 0.1	2.77	518.7
CF_0.5_-PI_3-2_-ScD (Et)	0.189 ± 0.007	0.6 ± 0.5	6.0 ± 3.6	31.1 ± 0.3	2.70	512.6
CF_1.5_-PI_3-2_-ScD (Et)	0.195 ± 0.004	1.2 ± 0.4	7.4 ± 2.5	33.4 ± 0.2	2.60	—
CF_1_-PI_1-1_-AD	0.213 ± 0.006	—	13.9 ± 4.1	42.8 ± 0.2	2.18	483.7
CF_1_-PI_1-1_-FD	0.231 ± 0.010	—	17.5 ± 2.5	47.8 ± 0.2	3.42	452.5
CF_1_-PI_1-1_-ScD (CO_2_)	0.146 ± 0.007	—	0.8 ± 0.1	31.6 ± 0.1	1.62	476.2

“—”in the table: Not measured.

## Data Availability

The data that support the findings of this study are available from the corresponding authors upon reasonable request.
